# Enhancing wound healing with synergistic dual-drug electrospun roflumilast and L-arginine loaded PLA/PVA nanofibers through fabrication, optimization, and *in vivo* assessment

**DOI:** 10.1038/s41598-026-38086-6

**Published:** 2026-02-20

**Authors:** Samar A. Salim, Abdullah M. M. Elbadry, Esraa B. Abdelazim, Tasneem Abed, Marwa Mosaad Shakweer, Noura G. Eissa, Elbadawy A. Kamoun, Mahmoud Elsabahy

**Affiliations:** 1 Nanotechnology Research Center (NTRC), The British University in Egypt (BUE), El-Sherouk City, Cairo, 11837 Egypt; 2 Department of Pharmaceutics and Industrial Pharmacy, Faculty of Pharmacy, Galala University, New Galala, 43713 Egypt; 3 Badr University in Cairo Research Center, Badr University in Cairo, Badr City, Cairo, 11829 Egypt; 4 Department of Pathology, Faculty of Medicine, Badr University in Cairo, Cairo, 11829 Egypt; 5 Department of Pathology, Faculty of Medicine, Ain Shams University, Cairo, Egypt; 6 Department of Pharmaceutics, Faculty of Pharmacy, Zagazig University, Zagazig, 44519 Egypt; 7 Department of Chemistry, College of Science, King Faisal University, Al-Ahsa, 31982 Saudi Arabia; 8 Polymeric Materials Research Department, Advanced Technology and New Materials Research Institute (ATNMRI), City of Scientific Research and Technological Applications (SRTA-City), Alexandria, 21934 Egypt; 9 Department of Pharmaceutics, Faculty of Pharmacy, Assiut University, Assiut, 71526 Egypt

**Keywords:** Electrospinning, Dual-drug, Nanofibers, Roflumilast, L-arginine, Wound healing, PLA, Drug delivery, Biotechnology, Chemistry, Drug discovery, Materials science, Medical research

## Abstract

Current trends in improved wound management emphasize the development of dual-drug-loaded electrospun nanofibrous scaffolds. Herein, electrospun dual-drug loaded PLA/PVA nanofibrous scaffolds, incorporating roflumilast, a selective phosphodiesterase-4 inhibitor with anti-inflammatory activity, and L-arginine, a precursor to nitric oxide with stimulating activity towards wound healing and tissue regeneration, were developed. A dual-spinneret electrospinning process enabled the co-loading of drugs in PLA and PVA phases. Scaffolds were characterized by SEM, FTIR, and XRD, showing consistent fiber morphology, and amorphous drug formation. FTIR analysis was performed to confirm drug–polymer compatibility and successful incorporation of roflumilast and L-arginine within the PLA/PVA nanofibrous matrix. Swelling ratio analysis indicated controlled hydration behavior regarding polymer–drug composition, and dual-drug-loaded fibers exhibited maximum initial swelling (610%) and stabilization at ~ 240%. *In vivo* rat excision wound healing model showed that the dual-drug loaded nanofibers demonstrated enhanced wound healing with ~ 99.8% healing on day 14, which was significantly better, compared to nanofibers incorporating a single drug and control groups. Moreover, histological studies revealed the absence of residual granulation tissue and enhanced re-epithelialization in the dual-drug treated group. These results indicate that roflumilast and L-arginine co-loaded PLA/PVA nanofibers exhibit wound-healing and regenerative properties, offering a promising therapeutic platform for enhanced wound repair.

## Introduction

The skin is the largest organ in the body, essential for various functions including excretion, vitamin D synthesis, protection against pathogens, hydration maintenance, and temperature regulation. Severe dermal injuries may be life-threatening, and the wound healing process exhibits numerous remarkable cellular and molecular mechanisms. The repair process involves several interactions among cells, growth factors, and cytokines to facilitate lesion closure^1,2^. Various types of wound dressings are currently available, including films, hydrogels, and electrospun nanofibers (NFs). These dressings create a moist, regulated environment that facilitates cellular growth and migration, leading to accelerated wound healing^3,4^. However, the painful removal of films and the fact that they apply mainly to non-highly exuding wounds are considerable limitations^5^. Hydrogels were also reported for management of wounds, yet insufficient adhesion and weak mechanical strength while maintaining moisture can be a concern^6^. Electrospun NFs have demonstrated promising therapeutic benefits such as wound dressings because of their mimicking to the natural extracellular matrix, which facilitate cell adhesion, migration, and tissue regeneration^7^. Additionally, electrospun NFs possess several advantages in wound healing applications due to their high surface area-to-volume ratio and interconnected porosity, and adjustable physicochemical properties^8^. Electrospinning is commonly utilized to fabricate NFs and adjusting electrospinning parameters determines the characteristics of the resultant fibers. Nanofibrous scaffolds can be synthesized using either natural polymers (e.g., collagen, chitosan, and hyaluronic acid), synthetic polymers (e.g., polylactide (PLA), and polyvinyl alcohol (PVA))^9^, or both^10,11^. PVA is a biodegradable synthetic polymer widely used in various applications, including wound dressings, due to its excellent hydrophilicity, biocompatibility, affordability, and crosslinking potential^12,13^. PVA is effective as a wound dressing material *via* maintaining a moist wound environment, enhancing the physical characteristics of the dressing, and accelerating the rate of wound healing^13^. Polylactic acid, also known as polylactide (PLA), is a highly biocompatible and biodegradable synthetic polymer approved by FDA for human use and extensively employed in pharmaceutical and biomedical industries, including tissue engineering, surgical suture fabrication, regenerative medicine, and wound healing^14–17^. PLA-based biomaterials facilitate wound healing by promoting wound area regeneration, collagen formation, angiogenesis, and controlling infection^18^. Incorporation of various natural or synthetic cargoes that possess wound healing properties into nanofibrous scaffolds have demonstrated significant improvement in capacity of tissue healing and regeneration^19^. Roflumilast is a selective phosphodiesterase-4 (PDE4) inhibitor with anti-inflammatory properties^20^. It is indicated for patients with severe chronic obstructive pulmonary disease associated with chronic bronchitis and a history of exacerbations to minimize the risk of further exacerbation. Additionally, it was approved by FDA as a topical cream for plaque psoriasis treatment in July 2022^21,22^. The inhibition of PDE4 leads to elevated intracellular levels of cyclic adenosine monophosphate (cAMP), subsequently reducing pro-inflammatory cytokines such as IL-6, IL-8, and TNF-α, thereby diminishing the recruitment and activation of inflammatory cells^23,24^. Additionally, cAMP controls the migration and proliferation of human keratinocytes by accelerating the re-epithelialization of wounds^25,26^. L-arginine is an endogenous amino acid produced predominantly as a metabolic product of the urea cycle. It is essential for making proteins, urea, creatine, proline, and nitric oxide (NO). The body uses arginine to produce NO, glutamate, and prolamins, which perform various regulatory functions^27,28^. L-arginine has many roles in accelerating wound healing through several biochemical pathways. It promotes blood vasodilation and angiogenesis as a precursor molecule to NO. This increases oxygen and blood delivery to the damaged tissues^29^. L-arginine assists in collagen synthesis by stimulating the production of proline and hydroxyproline, which are essential for extracellular matrix formation and tissue integrity^30^. It also enhances the immune system by stimulating T-cell and macrophage functions and cell proliferation by converting to polyamines *via* the ornithine pathway^31^. Additionally, L-arginine helps the body secrete growth hormone and insulin-like growth factor 1, which are both essential for protein synthesis and tissue growth. This identifies L-arginine an essential amino acid throughout all stages of wound healing^32,33^.

In this study, we developed roflumilast-loaded PLA and L-arginine-loaded PVA nanofibers through a dual-spinneret electrospinning technique. The PLA/PVA blend was chosen to combine the hydrophilicity, swelling capacity, and moisture-retention capabilities of PVA with the advantageous mechanical strength, biodegradability, and prolonged drug release characteristics of PLA, all of which are critical for successful wound healing applications^34^. The two polymers’ complementing physicochemical properties allow for the creation of nanofibrous scaffolds that offer tissue regeneration-promoting hydration and structural integrity^35,36^. The aim is to investigate the synergistic effect of these bioactive components on enhancing wound healing rate by combining their diverse pharmacological properties.

## Materials and methods

### Materials

Roflumilast was generously gifted by Al Andalous Pharmaceutical Industries (Egypt). Polylactic acid (PLA) (Ingeo™ 6202D) was purchased from NatureWorks LLC (USA), while L-arginine and chloroform were purchased from Merck (Germany). Polyvinyl alcohol (PVA) was obtained from Alpha Chemika (Mumbai, India).

### Methods

#### Preparation of electrospun nanofibrous scaffolds

Two separate polymer solutions were used as the base for dual-spinneret electrospinning. The first solution, PLA was dissolved into chloroform at a concentration of 15% (*w*/*v*) by gradually adding the precisely weighed polymer to the solvent under room-temperature and magnetic stirring until a homogeneous, clear solution was obtained. For the second solution, PVA was dissolved in distilled water at 10% (*w*/*v*) concentration under 60 °C temperature and stirring conditions to ensure complete dissolution. Drug-loaded solutions were then prepared by adding the active compounds to the polymer matrices through the following procedures: roflumilast powder was added to the PLA solution at 0.5% (*w*/*v*) and stirred to become homogeneously dispersed. L-arginine powder was added to the PVA solution at 0.5% (*w*/*v*) and was blended until fully dissolved. Roflumilast was added to the PLA solution because it dissolves effectively in chloroform, while L-arginine was incorporated into the PVA solution due to its water solubility. This approach allows us to achieve high solubility for both substances. The selected concentration was based on previously published studies that show effective and safe loading of L-arginine^31^and roflumilast in topical and electrospun delivery systems^37^. These pilot studies revealed that at concentrations about 0.5% (w/v), maintain the solution spinnability, fiber uniformity, and scaffold integrity, and suboptimal in vivo wound healing performance. Four nanofibrous scaffolds were prepared as follows and their composition and concentrations are demonstrated in Table 1. **F1**: (PLA/PVA), drug-free control formulation; both spinnerets contained pure polymer solutions, PLA solution and PVA solution. **F2**: (PLA-Roflumilast/PVA), 0.5% (*w*/*v*) roflumilast-loaded PLA solution blended with drug-free PVA solution. **F3**: (PLA/PVA-L-arginine), drug-free PLA solution blended with 0.5% (*w*/*v*) L-arginine-loaded PVA solution. **F4**: (PLA-Roflumilast/PVA-L-arginine), both solutions were drug-loaded, roflumilast in PLA and L-arginine in PVA. These combinations allowed direct comparison of drug-free, single-drug, and dual-drug-loaded scaffolds under the same electrospinning conditions.

#### Electrospinning process

**Fig. 1:**
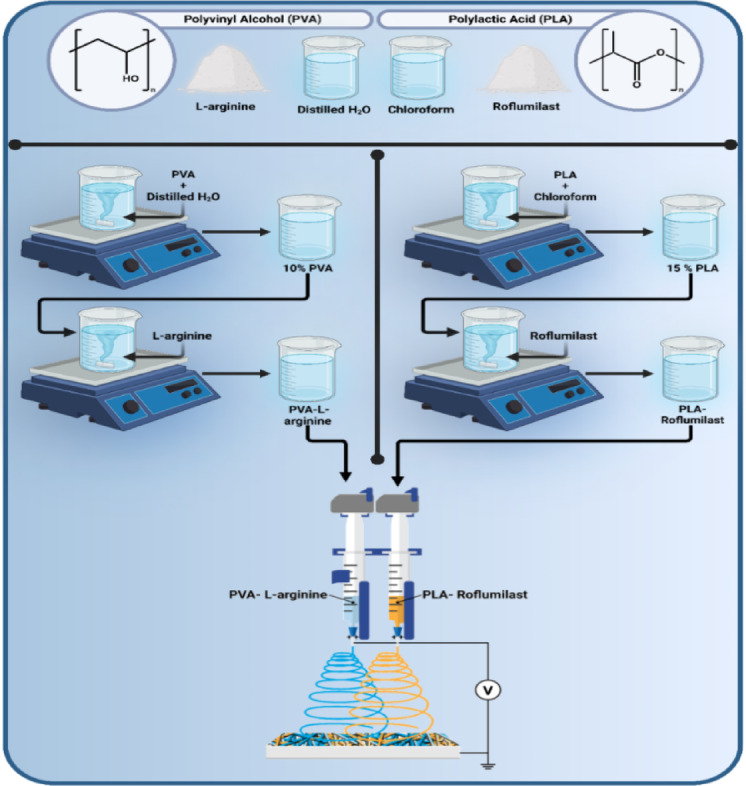
Procedures for fabrication of nanofibrous scaffolds.

Nanofiber scaffolds were fabricated using a dual-spinneret electrospinning apparatus (NANON-01 A, MECC, Japan) (Fig. 1). Polymer solutions were loaded in a 5 mL syringe with a 22G stainless steel needle and connected to individual syringe pumps to spin simultaneously. Solutions were electrospun onto a flat plate collector wrapped with aluminum foil. Electrospinning parameters, including applied voltage, tip-to-collector distance, and collector width, were also optimized for even fiber deposition (applied feed rate of 0.5 mL/h, spinneret speed of 100 rpm, spinneret width of 150 mm at a distance of 15 cm, and the applied voltage of 26.5–28.5 kV). All the protocols were carried out in a controlled environment (relative humidity: 41.0 ± 2.0%). After electrospinning, the nanofiber matrices were carefully peeled from the collector and transferred to a vacuum chamber overnight to remove all remaining chloroform and water before characterization.

**Table 1:** Composition and concentrations of the components of the nanofibrous scaffolds.

Code	Composition	PLA (%, w/v)	PVA (%, w/v)	Roflumilast (%, w/v)	L-arginine (%, w/v)	Voltage (kV)
**F1**	PLA/PVA	15	10	-	-	26.5
**F2**	PLA-Roflumilast/PVA	15	10	0.5	-	28.5
**F3**	PLA/PVA-L-arginine	15	10	-	0.5	28.5
**F4**	PLA-Roflumilast/PVA-L-arginine	15	10	0.5	0.5	28.5

### Characterizations of nanofibers

#### Scanning electron microscopy

The surface morphology of the blended NFs was examined using a field emission environmental scanning electron microscope (FE-SEM) (Quattro S, Thermo Scientific, USA) operated at an acceleration voltage of 15 kV. The samples were observed without any additional coating to ensure accurate imaging and analysis. Further image processing was performed using ImageJ software^38^.

#### Fourier transform-infrared spectroscopy

The chemical composition and molecular structure of the NFs scaffolds were analyzed using Fourier Transform Infrared (FT-IR) Spectroscopy. Measurements were conducted using an FT-IR spectrometer (Bruker Vertex 70, Germany), covering the spectral range of 4000–400 cm⁻¹ to identify characteristic functional groups and molecular interactions^39^.

#### X-ray diffraction analysis

X-ray diffraction (XRD) diffractometer (Malvern PANalytical, England, UK) with Cu Kα radiation was used to examine the crystalline structure of the electrospun NFs. The diffraction patterns were recorded as intensity versus 2θ, with an angular range of 5° to 80°, a step increment of 0.02°, and a scanning time of 0.5 s per step.

#### Study of swelling ratio (%)

The swelling ratio of the NFs was determined based on their ability to absorb water. Pre-weighed NFs (W_d_) were immersed in 20 mL of deionized water at room temperature for 6 h. After incubation, the NFs were reweighed (W_e_) in their hydrated condition, and the swelling ratio was estimated at specified time intervals using the following equation:

1 $$\:Swelling\:ratio\:\left(\mathrm{\%}\right)=\left(\frac{\mathrm{W}\mathrm{e}-\mathrm{W}\mathrm{d}}{\mathrm{W}\mathrm{d}}\right)\times\:100\:$$

A volume of solvent of 20mL was used in order to create sink conditions and facilitate consistent measurements for swelling under standardized in vitro conditions. This volume was used in order to prevent rapid saturation of the medium, thus facilitating comparisons with respect to swelling characteristics among formulations, rather than attempting to match physiological exudate volume in a wound.

### *In vivo* wound healing assay

The *in vivo* experiments were ethically approved by Badr University in Cairo-Institutional Ethical Committee No. (BUC-IACUC-241020-115). All methods were carried out in accordance with relevant guidelines and regulations. Twenty male *Wistar* rats (230–250 g) were obtained from the animal house at Badr University in Cairo. The rats were housed in separate polycarbonate cages at 26–28 °C, and a 12-h dark cycle with standard water and food.

A wound healing assay was conducted using twenty male Wistar rats. All animals were anesthetized with ketamine (90 mg/Kg) injection, then the back was shaved, and a biopsy punch was used to create 1 cm^2^ skin wounds on the back of the rats^40^. To avoid individual differences, rats were randomly divided into **Group I**, which received no treatment, and served as a negative control. **Group II** was treated with F1 (PLA/PVA nanofiber) and served as positive control. **Group III** was treated with F2 (PLA-Roflumilast/PVA nanofiber), **Group IV** was treated with F3 (PLA/PVA-L-arginine nanofiber), and **Group V** was treated with F4 (PLA-Roflumilast/PVA-L-arginine nanofiber) (*n* = 4). Wounds in all groups were covered with sterile gauze to avoid infection. Changes in wound area were measured after 3, 7, 10, and 14 days, the wound area that remained exposed was measured, and the percentage of wound healing was calculated using Eq. (2)^11^:

2 $$\:Wound\:healing\:\%=\frac{{A}_{i}-{A}_{d}}{{A}_{i}}\times\:100$$

A_d_ and A_i_ are the wound areas on the specified day and day zero, respectively. The rats in the groups were sacrificed on day 14 by anaesthetizing the rats with intramuscular injections of xylazine (15 mg/kg of body weight) and ketamine (60 mg/kg of body weight). To preserve the skin samples for later histological analysis, they were immediately placed in 10% formalin.

### Histopathological assessment

The wound sites in the four groups were biopsied. Specimens fixed in 10% phosphate-buffered formalin and 3–5 μm sections were prepared and stained with hematoxylin and Eosin stain (H&E). The degree of wound healing was assessed by both granulation tissue and fibrosis as follows:

- Residual granulation tissue indicates delayed wound healing.
- Complete fibrosis indicates complete wound healing.

## Results and discussion

### Construction of dual electrospun nanofibrous scaffolds

Multiple nanofiber scaffolds were produced under varying electrospinning conditions to enhance fiber morphology, uniformity, and layer integrity. The F1 scaffold consisted of a 15% (w/v) PLA solution combined with a 10% (w/v) PVA solution, which served as the standard scaffold and exhibited optimal fiber morphology and consistency, resulting in smooth and uniform nanofibers. The F2 scaffold was developed with PLA-Roflumilast/PVA, functioning as a single drug-loaded scaffold with a Roflumilast loading concentration of 0.5%. In contrast, the F3 scaffold was created by incorporating 0.5% L-Arginine into a 10% PVA solution, resulting in a PLA/PVA-L-Arginine scaffold. The F4 scaffold integrated both fixed concentrations (0.5%) of each Roflumilast and L-Arginine into a single formulation, labeled as PLA-Roflumilast/PVA-L-Arginine. All scaffolds were electrospun onto a flat plate collector, showcasing randomly oriented fibers with consistent thickness and smooth surfaces.

### SEM investigation

**Fig. 2:**
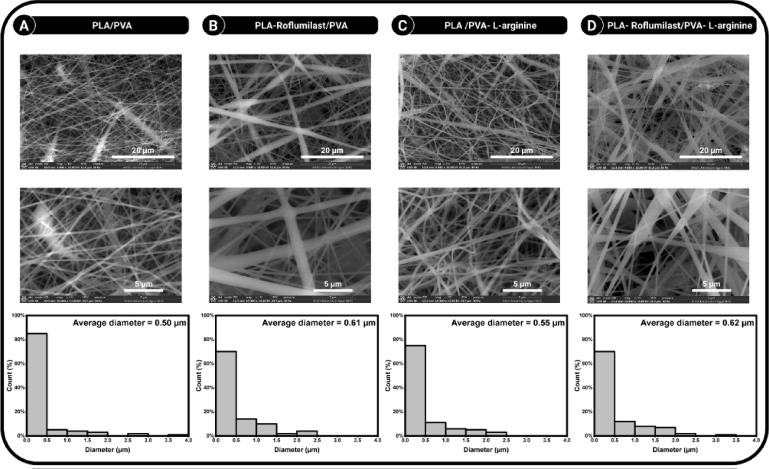
SEM images of **(A)** (PLA/PVA), **(B)** (PLA-Roflumilast/PVA), **(C)** (PLA/PVA-L-arginine), and **(D)** (PLA-Roflumilast/PVA-L-arginine) NFs. The Lower panel represents diameter distribution histograms of the nanofibers.

The electrospun nanofibrous scaffolds displayed different morphological and structural characteristics, affirming the effect of drug incorporation and polymer composition on fiber architecture. Figure 2A demonstrates that the PLA/PVA blend produced smooth, bead less fibers with a mean diameter of 0.50 μm and pores area 9.04%. This indicates that the selected polymeric system was electro-spinnable and produced nanoscale fibers suitable for biomedical applications such as drug delivery and tissue engineering^36^. Constant jet stability and optimum process conditions are also supported by the homogenous distribution observed in the histogram. Incorporating roflumilast into the PLA/PVA matrix (Fig. 2B) resulted in a notable increase in fiber diameter to 0.61 μm and pores area to 10.8% and a broader distribution compared to the control sample that might be attributed to the change in solutions’ charge density, conductivity, and viscosity during drug loading^41^. The absence of bead formation suggests that the electrospinning process was steady enough to accommodate homogeneous encapsulation inside the fibers. Moreover, introducing L-arginine into the PLA/PVA system (Fig. 2C) also affected fiber morphology, having a mean diameter of 0.55 μm and pores area 11.8%. The introduction of L-arginine, a cationic amino acid, may have improved solution conductivity, allowing thinner fiber generation than the roflumilast-loaded fiber matrix^42^. The NFs incorporating both roflumilast and L-arginine had a mean thickness of 0.62 μm, small pores area 4.8% and a moderately broad distribution (Fig. 2D). The synergistic action between the drug and the charged biomolecule would most likely create competing effects on conductivity and viscosity, leading to thicker fibers^42^. Yet, the morphology was bead-free and uniform, demonstrating that the system could tolerate complex co-loading without defects in processing. These findings support the implication that incorporated drugs can contribute to the electrospun fiber properties. In F2, roflumilast loading increased the mean fiber diameter, whereas adding L-arginine (a cationic amino acid) in F3 likely increased solution conductivity, stabilizing the jet and limiting diameter growth. The observed submicron diameters of fibers are particularly suitable for high surface area to volume ratios to enable efficient drug release and biocompatible interaction with biological tissues.

### FTIR analysis

**Fig. 3:**
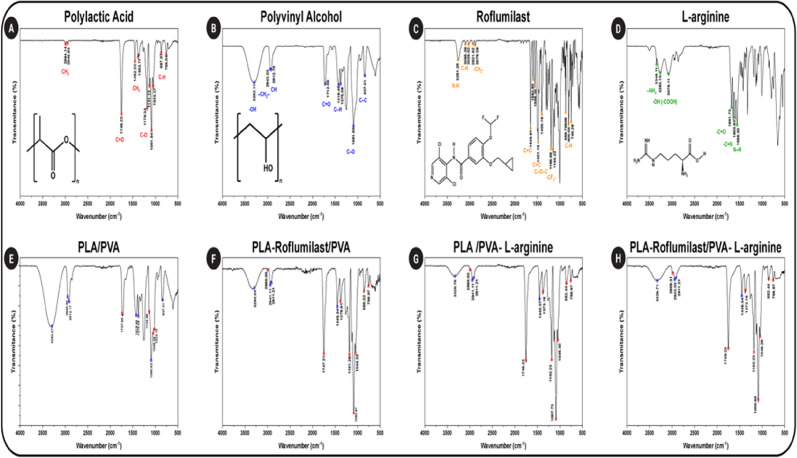
FTIR spectra of **(A)** PLA, (B) PVA, **(C)** Roflumilast, **(D)** L-arginine, **(E)** (PLA/PVA), **(F)** (PLA-Roflumilast/PVA), **(G)** (PLA/PVA-L-arginine), and **(H)** (PLA-Roflumilast/PVA-L-arginine) NFs.

FTIR spectroscopy was utilized to confirm the successful fabrication of PLA/PVA NFs and determine the loading of roflumilast and L-arginine into the electrospun scaffolds. FTIR is a convenient analytical method for identifying functional groups and monitoring possible interactions among polymers and active pharmaceutical ingredients. The pure polymers (PLA and PVA), pure drugs (roflumilast and L-arginine), and the respective nanofiber composites were compared systematically in terms of spectra. PLA had characteristic absorption bands at ν ~ 1750 cm⁻¹ (ester group C = O stretching), ν ~ 1180–1085 cm⁻¹ (C–O stretching vibrations), the peaks at ν 755 and 867 cm^− 1^ (C–H-bond stretches), and ν ~ 1450–1380 cm⁻¹ (CH₃ bending) (Fig. 3A). These peaks reflect the ester backbone and confirm the semi-crystalline nature of PLA^43,44^. PVA exhibited a broad, strong band at ν ~ 3300 cm⁻¹ for O–H stretching typical of hydrogen bonding within the polymer and a shoulder at ν 1142 cm⁻¹ typical of C–O stretching. Peaks at ν 2943 cm⁻¹ and 2912 cm⁻¹ correspond to the stretching vibrations of the methylene (–CH_2_–) and alkyl (C–H) groups, respectively (Fig. 3B). A peak at ν 1712 cm⁻¹ indicates the carbonyl (C = O) stretching vibration, likely from acetate groups. The peaks at ν 1419 cm⁻¹ and 1375 cm⁻¹ are associated with the bending vibration of the methylene (CH_2_) group and the deformation vibration of C–H, respectively. Furthermore, the peak at ν 837 cm⁻¹ is attributed to C–C stretching vibrations. The strong hydrophilic character of PVA is evident in these peaks^45–47^. The spectrum of roflumilast (Fig. 3C) shows several characteristic vibrational peaks that are indicative of its molecular structure. The absorption at ν 3261 cm⁻¹ corresponds to N-H stretching vibrations, while the peaks at ν 3095 and 3029 cm⁻¹ are attributed to C-H stretching in the aromatic ring and cyclopropyl group. The aliphatic -CH₂- group exhibits symmetric and asymmetric stretching at ν 2921 and 2879 cm⁻¹. A notable peak at ν 1649 cm⁻¹ corresponds to C = O stretching in the amide moiety, and the bands between ν 1594 and 1400 cm⁻¹ are associated with C = C stretching in the aromatic ring. Additionally, the peak at ν 1196 cm⁻¹ is due to symmetrical C–O–C stretching within the cyclopropyl group, while the absorption at ν 1155 cm⁻¹ reflects asymmetric stretching of -CF₂- group. The peaks at 869, 808, and 748 cm⁻¹ indicate C-H out-of-plane bending in the aromatic ring^48,49^. The L-arginine peaks (Fig. 3D) identified at ν 3263 cm⁻¹ and 3346 cm⁻¹ correspond to the stretching vibrations of the guanidine and primary amine groups, respectively. The peaks at ν 3076 cm⁻¹, 1681 cm⁻¹, 1603 cm⁻¹, and 1556 cm⁻¹ are attributed to the stretching vibrations of -OH group (from -COOH), -C = O, and -C = N bonds, alongside the bending vibrations of the N–H bond^50^. PLA/PVA blend spectrum (Fig. 3E) retained the main functional peaks of the two polymers, like the ester C = O stretching of PLA and the broad hydroxyl band of PVA. The overlap of this kind suggested compatibility of the two polymers at the molecular level and the ability to form a stable blended fiber matrix. The broadening of the O–H stretching peak suggested hydrogen bonding interactions between PLA carbonyl groups and PVA hydroxyl groups, potentially leading to enhanced miscibility^36^. The addition of roflumilast into PLA/PVA fibers (Fig. 3F) introduced additional signals to the aromatic moieties and amid functional groups of the drug. Even though these peaks overlapped partly with the polymer background, changes in intensity and minor shifts in carbonyl stretching bands could be observed. These reflect probable hydrogen bonding or dipole–dipole interactions between the functional groups of roflumilast and the polymer chain, maintaining drug dispersion but without any chemical incompatibility^48,49^. Similarly, PLA/PVA fibers with L-arginine (Fig. 3G) showed spectral intensities attributed to the amino acid, specifically in the ~ 1650 cm⁻¹ region (NH₂ bending) and within the broad ν 3200–3400 cm⁻¹ region. These absorptions blended with the PVA hydroxyl and PLA carbonyl regions are characteristic of hydrogen bonding between L-arginine functional groups and the polymer matrix. This interaction should stabilize drug loading as well as may influence drug release kinetics^50^. In dual-drug loaded formulation (PLA-Roflumilast/PVA-L-arginine) (Fig. 3H), combined features of both bioactive molecules were evident. The presence of both aromatic and amine/carboxylate signals, along with the polymer peaks, confirmed effective co-encapsulation. Interestingly, no new typical peaks were seen, and no substantial shifts indicated chemical degradation or incompatibility. Instead, the broadening of peaks and slight intensity changes in O–H and C = O regions indicate non-covalent physical interactions that hold the composite framework together. Absence of new chemical bonds shows that both roflumilast and L-arginine are physically entrapped, rather than chemically conjugated, within the nanofiber matrix. Physical entrapment is advantageous for drug release since it preserves the bioactivity of the loaded drugs and allows controlled release modulated by diffusion and degradation of the polymer^51^. The implied spectral overlaps and hydrogen bonding interactions also indicate a stable drug–polymer network, preventing the risks of the drugs’ crystallization or phase separation within the fibers^52^. The FTIR findings confirm that PLA/PVA nanofibers are viable carriers for hydrophobic (roflumilast) as well as hydrophilic (L-arginine) drugs. The drug-polymer compatibility assures fiber structural integrity, whereas the physical interactions found are most likely to ensure drug homogeneity of distribution and reproducible release kinetics.

### XRD analysis

**Fig. 4:**
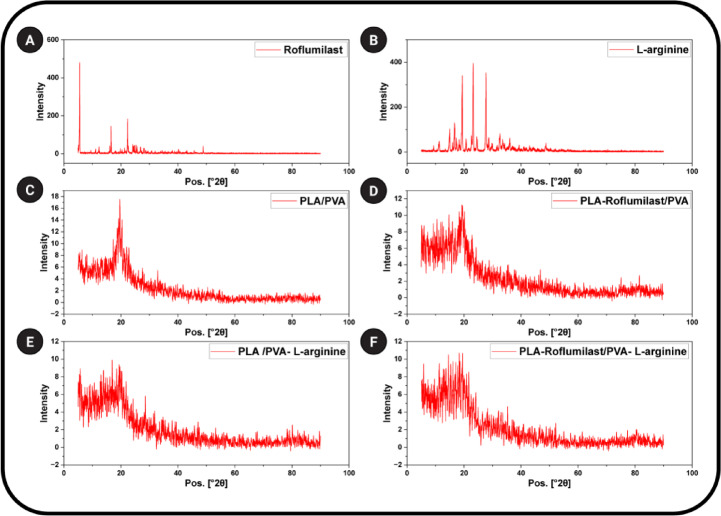
XRD patterns of **(A)** Roflumilast, **(B)** L-arginine, **(C)** (PLA/PVA), **(D)** (PLA-Roflumilast/PVA), **(E)** (PLA/PVA-L-arginine), and **(F)** (PLA-Roflumilast/PVA-L-arginine).

The XRD analysis shows the diffraction patterns of the tested samples, which reflects their structural and phase properties. XRD pattern of roflumilast and L-arginine show a series of sharp and intense diffraction peaks, suggesting a highly crystalline structure (Figs. 4A and B). Roflumilast exhibited typical peaks at 2θ = 5.55°, 11.15°, 12.31°, 16.57°, 22.35°, 24.13°, and 48.83°, which agree with reported crystalline patterns of the compound^53^. L-arginine exhibited strong peaks at 2θ = 9.31°, 11.31°, 15.01°, 16.65°, 19.37°, 23.21°, 27.69°, and 32.01°, in accordance with the literature^54,55^. The presence of such typical peaks highlights the good packing architecture of molecules and periodicity in crystalline solids. However, the XRD patterns of the electrospun polymer-based NFs PLA/PVA, PLA-Roflumilast/PVA, PLA/PVA-L-arginine, and PLA-Roflumilast/PVA-L-arginine (Fig. 4C and F) demonstrate a broad amorphous rather than sharp crystalline peaks. This transition to an amorphous pattern indicates that the electrospinning process effectively incorporated roflumilast and L-arginine into the polymer matrix in a non-crystalline form. The transformation of the crystalline drugs into an amorphous state through electrospinning may enhance their dissolution rates and improve bioavailability^56,57^. The ternary formulation with PLA/PVA, roflumilast, and L-arginine (Fig. 4F) did not exhibit crystalline peaks for the drug or amino acid. Instead, the trace showed an amorphous polymeric blend. The complete absence of crystalline peaks indicates that the two active molecules were thoroughly dispersed at the molecular level in the polymeric nanofibers. Amorphous form normally exhibits higher dissolution rates due to greater free energy and a lack of lattice energy barriers. Furthermore, uniform molecular dispersion in the PLA/PVA matrix can prevent recrystallization, giving rise to long-term formulation stability^58^.

### Study of swelling ratio (%)

**Fig. 5:**
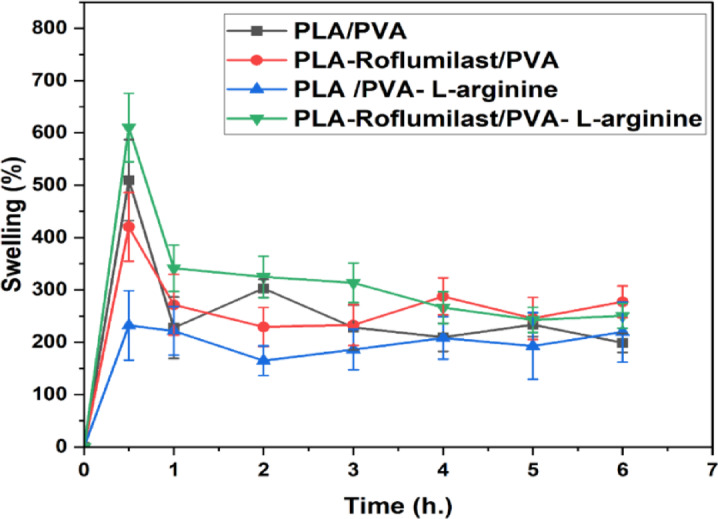
Swelling ratio (%) of the nanofiber scaffolds, PLA/PVA (F1) (Square), PLA-Roflumilast/PVA (F2) (Circle), PLA/PVA-Roflumilast (F3) (Triangle), and PLA-Roflumilast/PVA-Roflumilast (F4) (Inverted triangle).

Swelling properties of the nanofibrous wound dressings also have important clinical applications because they directly correlate to the absorbing capacity of the exudates in the wound and the creation of a moist microenvironment to encourage cell migration, angiogenesis, and re-epithelialization. Excessive swelling could result in the instability of the structure in addition to maceration of the wounds, whilst a low-swelling ratio could cause the adverse effects of poor exudates management^7^. Additionally, the swelling nature of electrospun nanofiber matrices is a critical parameter that controls their drug-releasing ability and hydration stability^59^. The swelling curves of PLA/PVA, PLA-Roflumilast/PVA, PLA/PVA-L-arginine, and PLA-Roflumilast/PVA-L-arginine nanofibers in a period of 6 h at room temperature are illustrated in Fig. 5. The findings exhibit how the water-soluble (PVA and L-arginine) and water-insoluble (PLA and Roflumilast) mixtures control the swelling kinetics and stabilization of the nanofibers. The control sample of PLA/PVA exhibited a strongly high initial swelling of ~ 510% during the first hour and slowly reduced to ~ 200%. This is because of the dissolution of PVA to some extent, which is extremely hydrophilic and highly water-absorbent, while the hydrophobic nature of PLA hinders excessive swelling^60,61^. The biphasic appearance observed by initial rapid swelling and stability is common in polymer blends where the amorphous water-soluble phase is accountable for primary hydration and the crystalline or hydrophobic phase is responsible for long-term structural stability^62^.

Roflumilast addition in F2 reduces relative swelling capacity, compared to PLA/PVA (F1) with a maximum swelling of ~ 420% at 1 h, stabilizing to ~ 220%. Roflumilast, being poorly water soluble, would tend to enhance the structural integrity of the NFs to decrease water diffusion channels. As demonstrated in previous research, loading of hydrophobic drug in electrospun fibers inhibited water uptake and altered swelling responses^63^. Moreover, F3 (PLA/PVA–L-arginine) exhibited the lowest swelling overall (~ 220% at 1 h, stabilizing near ~ 190%). Although L-arginine is hydrophilic, its rapid leaching forms drainable pores and its ionic/hydrogen-bond interactions with the polymer phase restrict chain relaxation, thereby reducing bound-water uptake at equilibrium^64^. In contrast, F4 (PLA-Roflumilast/PVA-L-arginine) showed the highest initial swelling (~ 610% at 1 h), driven by the hydrophilicity and osmotic action of the PVA/L-arginine phase, as soluble components dissolved, swelling declined to a moderate plateau (~ 240%), with the hydrophobic PLA/roflumilast framework preserving structural integrity. Thus, L-arginine can promote fast early hydration (F4 spike) while limiting water retention at later times (low plateau), depending on formulation and morphology^65^.

Dissolution of the components over a period caused a reduction in swelling, but the presence of PLA and roflumilast preserved matrix integrity and prevented over-collapsing of the structure. This balance between instant hydration and long-term stability is a desirable character in drug delivery because it allows for initial burst release followed by subsequent duration of sustained release.

Swelling profiles showed that drug loading and polymer composition significantly impact nanofiber hydration and structural stability. As demonstrated by the dual-drug loaded system, excellent swelling capacity can improve the release of amorphous roflumilast to increase bioavailability. Conversely, lower swelling in the L-arginine-loaded system shows controlled release capability with minimal burst effect. These results show that swelling response can be fine-tuned with high specificity to obtain a target drug release profile, with design flexibility for multifunctional therapeutic delivery systems.

### *In vivo* assessment

**Fig. 6:**
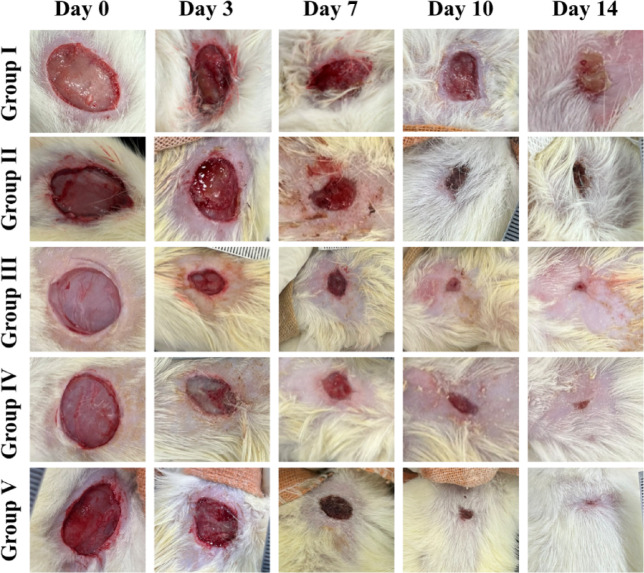
Representative photographic images of wounds for Group I (sterile gauze), Group II (treated with F1), Group III (treated with F2), Group IV (treated with F3), and Group V (treated with F4) on days 0, 3, 7, 10, and 14.

**Fig. 7:**
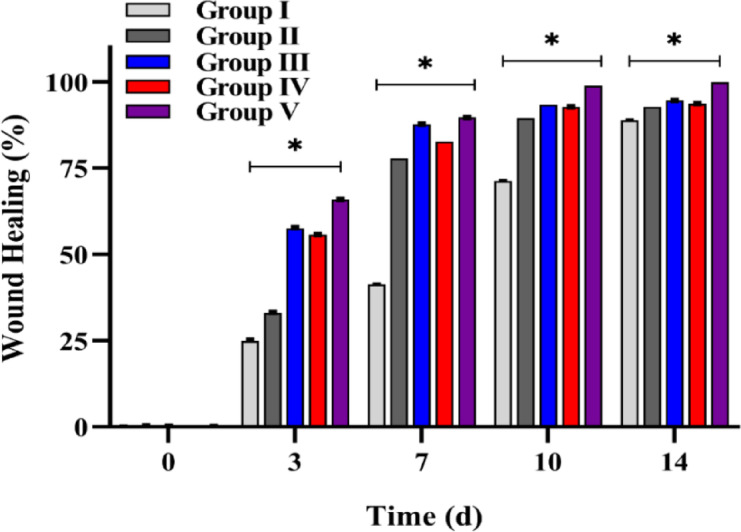
Bar diagram represents the % wound area after treating the wound with drug-loaded nanofibers and the control groups. ^*^ Statistical significance between Group V and Group I (*p* < 0.0001).

A wound model was developed to assess the wound healing capacity of the developed NFs. The wound images (Fig. 6) were analysed after 3, 7, 10, and 14 days of treatment. The wounds treated with F4 incorporating dual drugs (roflumilast and L-arginine) displayed excellent healing properties as compared to the control untreated group and the group treated with drug-free nanofiber (F1). In the control group, the wound healing rate reached 24.9 ± 0.5%, 41.2 ± 0.16%, and 71.2 ± 0.1% on the 3rd, 7th, and 10th days (Fig. 7), and large unhealed wounds were identified. The healing rate of wounds treated with F2 (roflumilast-loaded nanofibers), F3 (L-arginine-loaded nanofibers) and F4 (dual-drug loaded nanofibers) ~ 90% on the 10th day of treatment, with almost complete wound closure (99.8 ± 0.5%) demonstrated in case of group V treated with F4 on day 14, that can be attributed to the presence of roflumilast and L-arginine in the nanofibers. Accordingly, F4 NFs is a potential wound dressing that can expedite wound healing.

### Histopathology study

**Fig. 8:**
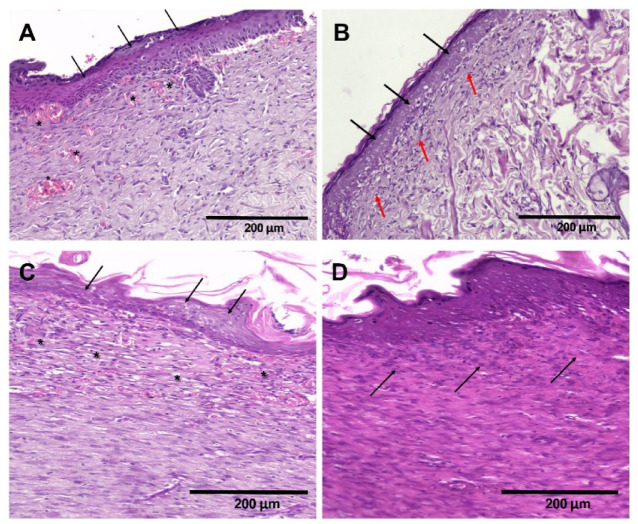
**(A)** Section from skin wound of group II showing residual granulation tissue in the upper dermis and newly formed blood vessels (*), indicating delayed wound healing. The covering is intact with complete epithelialization (arrows). H&Ex200 **(B)** Section from skin wound of group III showing fibrosis in the upper dermis with mild mononuclear inflammatory infiltrate (red arrows) and no residual granulation tissue. The epidermis shows complete epithelial closure (black arrows). H&Ex200 **(C)** Section from skin wound of group IV showing residual granulation tissue in the upper dermis and newly formed blood vessels (*), indicating delayed wound healing. The covering is intact with complete epithelialization (arrows). H&Ex200 **(D)** Section from skin wound of group V shows complete wound healing evidenced by complete fibrosis in the dermis (arrows) with no residual granulation tissue. H&E x200.

(Fig. 8A) illustrates a section from the skin wound of Group II revealing residual granulation tissue in the upper dermis and newly formed blood vessels, which indicate delayed wound healing. The covering is intact with complete epithelialization. (Fig. 8B) reveals a section from the skin wound of group III (treated with F2) showing fibrosis in the upper dermis with mild mononuclear inflammatory infiltrate and no residual granulation tissue. The epidermis shows complete epithelial closure. (Fig. 8C) depicts a section from the skin wound of group IV, where residual granulation tissue in the upper dermis and newly formed blood vessels are clearly observed, indicating delayed wound healing. The covering is intact with complete epithelialization. (Fig. 8D) reveals the section from the skin wound of group V showing complete wound healing, as evidenced by the complete fibrosis in the dermis with no residual granulation tissue. Group II has a similar appearance to Group IV, characterized by residual granulation tissue with minimal fibrosis. In contrast, Group III displays fibrous tissue without any granulation tissue. These results suggest that roflumilast has a more potent effect on wound healing compared to L-arginine. Combined treatment with roflumilast and l-arginine has a more effective response on the wound healing than each component alone, in the form of complete fibrosis with no residual granulation tissue.

## Conclusions

This study successfully demonstrated the development of dual-drug electrospun PLA/PVA NFs loaded with roflumilast and L-arginine as a drug-delivery multifunctional wound dressing system. The morphological and spectroscopical assays confirmed the polymer-drug matrix integrity, and XRD confirmed amorphous dispersion of the two active ingredients, an alteration that enhances dissolution and delays recrystallization. The swelling behavior also demonstrated the capacity of the dual drug loaded scaffold to promote rapid hydration without compromising the long-term structural integrity, a valuable characteristic for controlled drug release. Moreover, biological testing prioritized the therapeutic efficacy of the dual-drug system. *In vivo* studies confirmed the enhanced rate of wound closure, ~ 99.8% healing on day 14, which was significantly higher compared to single-drug and control groups. Histopathological studies also demonstrated the wound healing properties of the dual drug loaded NFs evidenced by complete fibrosis, absence of granulation tissue, and enhanced re-epithelialization, confirming that the scaffold not only closed the wound but also enabled high-quality tissue formation. Collectively, these findings confirmed the potential delivery of an anti-inflammatory PDE-4 inhibitor and a nitric oxide precursor from a nanofibrous matrix. The system effectively integrates structural, physicochemical, and bioactive functionalities into a single platform with synergistic therapeutic impact. The possibilities extend beyond wound dressings, indicating a broadly applicable drug delivery method with potential for application to the broader fields of tissue engineering and regenerative medicine.

## Data Availability

The datasets used and analyzed during the current study are available from the corresponding author on reasonable request.
